# Empathy Moderates the Effect of Repetitive Transcranial Magnetic Stimulation of the Right Dorsolateral Prefrontal Cortex on Costly Punishment

**DOI:** 10.1371/journal.pone.0044747

**Published:** 2012-09-13

**Authors:** Martin Brüne, Dirk Scheele, Christine Heinisch, Cumhur Tas, Julia Wischniewski, Onur Güntürkün

**Affiliations:** 1 Research Department of Cognitive Neuropsychiatry and Psychiatric Preventive Medicine, Landschaftsverband Westfalen Lippe University Hospital, Ruhr-University Bochum, Bochum, Germany; 2 Department of Psychology, University of Bonn, Bonn, Germany; 3 Department of Biopsychology, Faculty of Psychology, Ruhr-University Bochum, Bochum, Germany; University of Regensburg, Germany

## Abstract

Humans incur considerable costs to punish unfairness directed towards themselves or others. Recent studies using repetitive transcranial magnetic stimulation (rTMS) suggest that the right dorsolateral prefrontal cortex (DLPFC) is causally involved in such strategic decisions. Presently, two partly divergent hypotheses are discussed, suggesting either that the right DLPFC is necessary to control selfish motives by implementing culturally transmitted social norms, or is involved in suppressing emotion-driven prepotent responses to perceived unfairness. Accordingly, we studied the role of the DLPFC in costly (i.e. third party) punishment by applying rTMS to the left and right DLPFC before playing a Dictator Game with the option to punish observed unfair behavior (DG-P). In addition, sham stimulation took place. Individual differences in empathy were assessed with the German version of the Interpersonal Reactivity Index. Costly punishment increased (non-significantly) upon disruption of the right – but not the left – DLPFC as compared to sham stimulation. However, empathy emerged as a highly significant moderator variable of the effect of rTMS over the right, but not left, DLPFC, suggesting that the right DLPFC is involved in controlling prepotent emotional responses to observed unfairness, depending on individual differences in empathy.

## Introduction

For centuries, philosophers have controversially debated the question whether human behavior is guided largely by selfish motives or altruism and empathy [1], [2]. In the past decades, neuroscientific approaches, based on evolutionary theories on reciprocal altruism among genetically unrelated individuals [3] and economic decision-making involved in cooperation and reciprocity [4], [5], have shed light on these issues. Research into the ecology of social rules and moral values have demonstrated that humans have evolved strong preferences for equity and reciprocity, and consequently reject rather than accept offers that are perceived as being unfair, even if foregoing a net gain of resources [6]. Moreover, when observing that others are intentionally disadvantaged, humans often punish unfair behavior at their own expense, regardless of the likelihood of receiving anything in return [7]. The underlying motivation of such costly acts (hence called “altruistic” or “costly” punishment) probably resides in the need to reinforce cooperation and to avoid inequity within social groups, suggesting that these behavioral tendencies were positively selected in human evolution [6].

Neuroimaging studies have revealed an extended neural network involved in economic decision-making including cortical midline structures such as the anterior cingulate cortex (ACC), the insula, the ventromedial prefrontal cortex (VMPFC), the DLPFC, as well as the caudate nucleus and the thalamus [8], [9]. The DLPFC has been known to be engaged in executive control, decision-making and inhibition of prepotent responses [10]. However, some opposing views exist about its function in economic decision-making. For example, Sanfey and colleagues [11] observed, using functional brain imaging, that the DLPFC as well as the insula were activated when subjects were confronted with unfair offers in an Ultimatum Game (UG). In the UG, a proposer (player A) suggests how to split a (virtual) amount of money (expressed in money units; MU); the recipients (player B) can either accept or reject the proposer's offers. In case the recipient rejects the offer, neither of the players receives anything, whereas upon acceptance the money is split as suggested. Interestingly, the activation of the right DLPFC was larger, relative to insula activation, when players in the position of a recipient in the UG accepted an unfair offer. The reversed pattern was found when an unfair offer was rejected, suggesting that the right DLPFC is involved in overriding an emotionally generated impulse to reject unfair offers [11]. Consistent with this interpretation, Greene et al. [12] found, in a functional brain imaging study, that judgments concerning the appropriateness of violations of personal moral standards, such as in (fictive) scenarios in which one person has to be sacrificed to save the lives of five others, were associated with DLPFC activation. Taken together, these studies support the view that emotional responses to unfairness, inequity or moral dilemmas can be overridden by the right DLPFC.

An alternative account of the role of the DLPFC in economic decision-making comes from studies using brain stimulation techniques. Repetitive transcranial magnetic stimulation (rTMS) is a tool that allows drawing firmer conclusions about the causal role of cortical areas in task performance by producing transient “virtual lesions” on the cortex surface [13]. In an experiment using repetitive transcranial magnetic stimulation (rTMS) over the right DLPFC, van 't Wout and colleagues [14] demonstrated that the inhibition of the right DLPFC led to a greater acceptance rate of unfair offers in an UG, compared to sham stimulation [14]. Moreover, in the sham condition, unfair offers were rejected faster than they were accepted and this effect was reversed after inhibition of the right DLPFC. Similarly, Knoch and colleagues [15] found, using rTMS or transcranial direct current stimulation (tDCS; [16]), that subjects rejected unfair offers less often after inhibition of the right DLPFC (but not after rTMS to the left DLPFC or sham stimulation). However, while subjects were affected in their fairness-related behavior, their fairness judgment was unchanged, which suggests that, while subjects were well able to recognize the proposers' unfairness, they were apparently unable to resist selfish motives of resource maximization [15], [16]. This finding is compatible with the interpretation that the function of the right DLPFC is to implement culturally acquired fairness norms by resisting or overriding selfish motives.

Indirect support for this view comes from a number of neuroimaging studies of the impact of psychopathy on the appreciation of fairness norms. For example, Rilling et al. [17] demonstrated that non-clinical individuals with low psychopathy scores activated the DLPFC more strongly compared to subjects with high psychopathy scores when making non-cooperative choices in an economic game that examined whether or not two players trusted each other and cooperated during recurrent social interactions, known as the iterated Prisoner's Dilemma game. Conversely, Glenn et al. [18] revealed that psychopathic individuals showed increased activity of the right DLPFC during decision-making involving personal moral dilemmas analogous to Greene et al.'s study [12]. These results suggest that psychopathic individuals activate the right DLPFC when overriding selfish motives (to act in morally acceptable ways), whereas subjects with low psychopathy scores need to activate the right DLPFC when behaving in an egocentric manner, that is, acting against their altruistic attitudes.

To study the role of the DLPFC in actively implementing fairness norms by punishing violations of social rules, a few studies have examined “altruistic” or “costly” punishment in a Dictator Game (DG) variant with the option to sanction or punish unfairness at the participant's own expense, henceforth referred to as DG-P. The DG differs from a UG in that the observed recipient (“B”) is forced to accept all offers of a proposer (“A”) [19] As third party observer, individuals who are willing to invest own resources to reinforce equity are regarded as strongly altruistically motivated [20], [8], because the pay-off of a third party in a DG-P is not directly affected as compared to that of a recipient (“B”) in a UG scenario. By and large, behavioral studies have shown that unfair proposals of less than 50 percent of the whole distributable amount of resources or MU run the risk of being punished by a third party [6]. In partial accordance with this finding, a recent functional brain imaging study showed differential activation of the left and right DLPFC during punishment of unfair behavior. Specifically, the left DLPFC was more strongly activated during third-party punishment, but not revenge-like behavior, whereas the right DLPFC was activated during trials involving only weak punishment. This result can be interpreted in a way that suggests that the right DLPFC exerts cognitive control to overcome selfish motives not to punish when punitive behavior is less rewarding [21]. Consistent with these findings, in a study, in which participants were asked to assess the responsibility and impose punishment to (virtual) perpetrators, the right DLPFC was involved in the evaluation of the responsibility of norm violators, suggesting that this brain region has a key role in the decision whether and when to punish or not to punish [22].

However, the afore-mentioned studies have some weaknesses such as lacking a control stimulation condition [14], pertaining only to the most unfair condition, but not the less unfair conditions [15], employing a between-subject approach [15] rather than a within-subject design, or disregarding individual differences in empathy or selfishness. With regard to the latter, Wischniewski and Brüne [23] reported that patients with borderline personality disorder who differed from controls on several psychological dimensions including empathy punished observed unfairness at similar rates compared to controls, but for diametrically opposite motivations. Specifically, whereas costly punishment in healthy subjects was associated with the dimension “agreeableness” of the Five-Factor Personality Inventory (NEO-FFI) and inversely correlated with Machiavellianism, the opposite pattern was found in patients with borderline personality disorder, suggesting that in borderline patients costly punishment was not driven by empathy [23]. Moreover, to our knowledge no study exist that has looked at task performance in a DG-P after inhibiting the DLPFC using rTMS.

Accordingly, we sought to examine the role of the right DLPFC in costly punishment using an rTMS protocol. In addition, we were interested in the question as to what extent responses were modulated by individual differences in empathy as measured by a widely used self-report questionnaire. We predicted that, if the right DLPFC is directly involved in suppressing prepotent emotional responses to unfairness, rTMS over the right DLPFC will lead to an increase in punishment in the DG-P. In addition, if empathy played a role in punishing unfair behavior, as was the case in our previous study in psychologically healthy subjects [23], the effect of rTMS should be moderated by individual differences in empathy scores. Alternatively, if the right DLPFC exerted control over selfish impulses, rTMS would lead to a reduction of one's willingness to punish observed unfairness.

## Results

### Dictator Game

**Figure 1:**
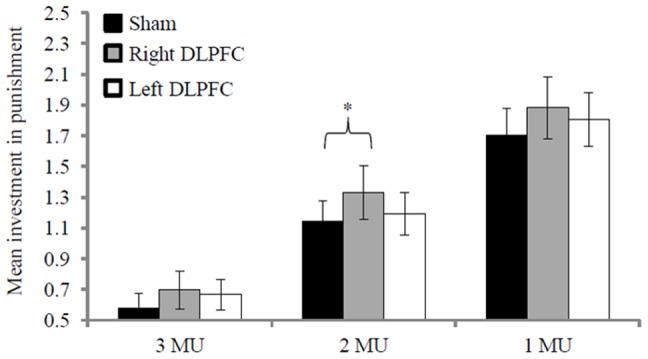
Mean punishment of unfair offers (3, 2, and 1 monetary unit; MU) in the Dictator Game with punishment option after TMS. Error bars represent standard errors. * p<.05.

No subject punished a proposer for a fair offer, but all subjects incurred personal costs to punish unfair money splits (*M* = 1.14 MU, *SD* = 0.56 MU) across rTMS conditions. Punishment varied across unfair offers in all stimulation conditions (*F*(1.63, 31.10) = 79.00, *p*<.01, η^2^ = .81) in that the punishment increased linearly to the extent that the fairness decreased, as exemplified here for the sham condition (*F*(1,19) = 104.94, *p*<.01, η^2^ = .85). The punishment for all unfair offers tended to be significantly different between rTMS conditions (*F*(2,38) = 2.66, *p* = .08, η^2^ = .12) (Figure 1). Importantly, when the SPQ empathy score was introduced into the equation as a covariate, the difference between the three stimulation conditions became significant (*F*(2,36) = 6.30, *p*<.01, η^2^ = .26). Simple planned contrasts revealed that this effect was due to greater punishment after stimulation of the right DLPFC (*M* = 1.30 MU, *SD* = 0.73 MU, *F*(1,18) = 8.71, *p*<.01, η^2^ = .33) compared to sham stimulation (*M* = 1.14 MU, *SD* = 0.56 MU). Punishment after stimulation of the left DLPFC (*M* = 1.22 MU, *SD* = 0.60 MU) was not significantly different from that after sham stimulation (*F*(1,18) = 1.69, *p* = .21, η^2^ = .09). Separate analyses showed the same pattern for each unfair condition, although the effect was strongest for the most unfair offer (Table 1).

**Table 1:** Repeated-measures ANOVA for the punishment investment across rTMS conditions with empathy as confound variable.

Offer	Mean punishment investment (SD)			*F*_(2,36)_	*p*	Partial η^2^
	Sham	Right DLPFC	Left DLPFC			
3 MU	0.58 (0.44)	0.70 (0.56)	0.67 (0.44)	5.36	<.01	.23
2 MU	1.14 (0.60)	1.33 (0.78)	1.19 (0.62)	4.00*	.04	.18
1 MU	1.71 (0.76)	1.88 (0.91)	1.80 (0.79)	5.81	<.01	.24
All unfair offers	1.14 (0.56)	1.30 (0.73)	1.22 (0.60)	6.30	<.01	.26

*Notes*. MU = monetary units; DLPFC = dorsolateral prefrontal cortex;

* df = 1.43, 25.43.

To corroborate this interpretation, we employed an analytic approach as proposed by Judd et al. [24], which allows testing directly the hypothesis that the empathy score acted as a moderator on the rTMS effect. Accordingly, we calculated the difference scores between rTMS and sham conditions (i.e. punishment after sham DLPFC stimulation minus punishment after right stimulation; punishment after left DLPFC stimulation minus punishment after right DLPFC stimulation; punishment after sham DLPFC stimulation minus punishment after left stimulation) and introduced these difference scores as dependent variables in three separate regression analyses where the SPQ empathy score served as predictor variable. Significant results were found for the sham minus right rTMS condition (β = .57, *t*(18) = 2.95, *p*<.01), and for the difference score between left and right rTMS (β = .44, *t*(18) = 2.08, *p* = .05), but not for the sham minus left rTMS regression (β = .26, *t*(18) = 1.12, *p* = .28). In other words, according to the moderator analysis, the effect of right rTMS versus sham and right versus left rTMS were successfully predicted by the SPQ empathy score, whereas the difference between sham and left rTMS was not moderated by empathy. Positive (i.e. >zero) *beta* values indicate that rTMS over the right DLPFC had a stronger effect in subjects with low empathy scores (that is, if punishment after rTMS to the right is greater than punishment in the sham condition, the difference between the two becomes negative, if punishment after rTMS to the right DLPFC is subtracted from punishment in the sham condition; accordingly, a positive *beta* value indicates that the difference is best predicted the lower the empathy score is).

**Figure 2:**
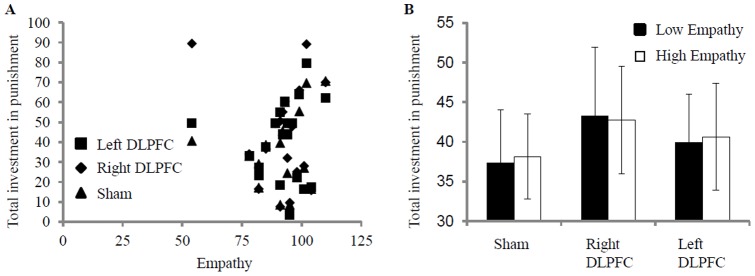
Association of empathy and punishment for the three rTMS conditions (A) and comparison of subjects scoring high or low on empathy (B). Error bars represent standard errors.

To investigate whether empathy was associated with punishment *per se*, independent of the stimulation, we calculated correlations between SFQ empathy scores and punishment in each rTMS condition. There were neither significant correlations (all *p*s>0.41; Figure 2 A) nor significant punishment differences (all *p*s>0.71; Figure 2 B) between subjects with low (below median) and high (above median) empathy scores.

### Digit Span

**Figure 3:**
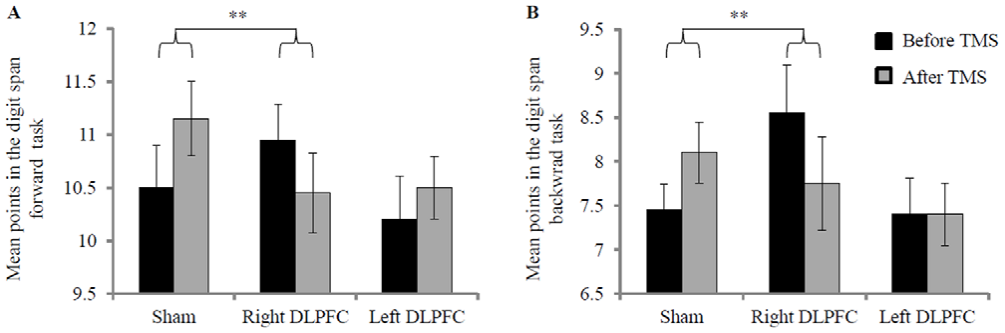
Mean scores in the digit span forward (A) and backward (B) task before and after TMS. Error bars represent standard errors. ** p<.01.

A repeated-measures ANOVA with stimulation (sham, left DLPFC, right DLPFC) and time (before and after TMS) as within-subject factors yielded a significant interaction between stimulation and time for DS forward (*F*(2, 38) = 3.22, *p* = .05, η^2^ = .15) as well as for DS backward (*F*(2, 38) = 5.50, *p* = .01, η^2^ = .22). Simple planned contrasts revealed that both interactions resulted from a significantly different time effect after sham stimulation compared to right DLPFC stimulation (DS forward: *F*(1, 19) = 11.81, *p*<.01, η^2^ = .39; DS backward: *F*(1, 19) = 12.69, *p*<.01, η^2^ = .40). Both interactions were disordinal, that is, DS forward performance improved from the first to the second measuring point in the sham and left DLPFC condition, but was impaired after inhibition of the right DLPFC. The same pattern was obtained for the DS backward performance, with the exception that the performance in the left DLPFC condition remained constant across the different measuring times (Figure 3). Neither the absolute DS scores (forward and backward) nor the differences between DS scores before and after stimulation were correlated with punishment in any condition (all *p*s>.12).

## Discussion

The present study sought to examine the contribution of the DLPFC in third-party punishment. We found that costly punishment increased upon the inhibition of the right DLPFC by rTMS, suggesting that the right DLPFC is involved in overriding prepotent emotionally aversive responses to perceived unfairness. Notably, the decision to punish or not to punish observed unfairness *per se* was not influenced by empathy. Instead, the rTMS effect on costly punishment was moderated by this personality trait.

Previous research has suggested that the right DLPFC is involved in controlling emotional responses to avoid inequity [14]. According to an alternative account, it has been proposed that the right DLPFC is necessary to override selfish impulses and to implement culturally acquired fairness norms [15]. A few studies into economic decision-making have applied either rTMS or tDCS over the right DLPFC. When subjects played an UG, they accepted more unfair offers upon inhibition of the right DLPFC compared to the left and sham stimulation [14]–[16]. Moreover, van 't Wout et al. [14] found that in the sham condition unfair offers were rejected faster than they were accepted and this effect was reversed after inhibition of the right DLPFC. Interestingly, in a recent study combining rTMS and neuroimaging Baumgartner et al. [25] found evidence for an altered connectivity between the DLPFC and the posterior VMPFC after rTMS over the right DLPFC, whereas the connectivity between the DLPFC and the insula remained unchanged. While these results corroborate the notion that rTMS may influence several parts of the neural circuitry underlying the rejection of unfairness, it does not rule out the possibility that the DLPFC controls emotional reactions to unfairness. For example, it could be that the DLPFC does not act upon the insula directly, but via other subcortical structures like the amygdala, which has recently been implicated in the processing of unfairness [26], [27].

To study the effect of rTMS on costly punishment directly, we introduced a DG-P, which has been assumed to measure altruistic attitudes more explicitly than the UG, because the participants' pay-off is not directly affected in the DG-P. Our findings suggest that the inhibition of the right, but not the left, DLPFC increased costly punishment. As in previous studies using the UG [15], [16], the rTMS effect was most evident in the most unfair conditions. However, a statistical model comparing right rTMS with left rTMS and sham stimulation was significant only at trend level. When empathy (as measured using the SPQ) was used as a covariate in the equation, the statistical equation modelling the effect of right DLPFC stimulation, left DLPFC stimulation and sham stimulation became highly significant. Moreover, individual differences in empathy moderated the rTMS effect, as revealed by regression analyses predicting differences between sham stimulation and rTMS over the right DLPFC, and between left and right rTMS, but not between sham stimulation and left rTMS.

Previous research has shown that individuals who are more “toughminded” demand higher shares than “tenderminded” individuals in a UG, while individuals with either high extraversion and emotional instability or low extraversion and greater emotional stability are more likely to reject unfair proposals, suggesting that the rejection of unfairness can be driven by aversive emotional responses against unfairness or “angry retaliation” [28]. Along the same lines, a recent study applying tryptophan depletion to lower the serotonin availability in the central nervous system demonstrated that this not only led to increased impulsivity, but, depending on the level of impulsivity, also to enhanced the rejection of unfair offers in a UG [29]. These findings are consistent with our previous study using a DG-P, which demonstrated that the motivation of patients with borderline personality disorder to engage in costly punishment is diametrically opposite to the motivation of unaffected controls [23]. That is, in borderline patients, punishment was correlated with Machiavellianism and inversely correlated with the personality trait “agreeableness” (as a measure of empathy), whereas in the control group, the opposite was the case.

In the present study, the regression coefficients suggest that the rTMS effect was stronger in individuals with low empathy scores compared to individuals with higher empathy scores. In line with the afore-mentioned studies, a tentative interpretation of this finding could therefore be that costly punishment, as reflected by an increase in investment to reduce the pay-off of an unfair proposer in a DG, is not necessarily a matter of empathetic concern for others, but perhaps linked to a prepotent emotional response to avoid inequity, which can be overridden by cognitive control mechanisms involving the right DLPFC. This interpretation is partially consistent with and may reconcile divergent interpretations of previous research using brain stimulation to manipulate behavior in the UG [14]–[16]. It is also compatible with neuroimaging studies in non-clinical individuals with high and low psychopathy scores [12], [17], [18], and with studies showing a differential activation of the DLPFC in costly punishment [22], [21].

The study has several limitations. First, similar to previous rTMS studies, the size of the effects caused by this brain stimulation technique was mild to moderate. This was not unexpected, however, due to the repeated-measures protocol examining within-subjects effects of rTMS. Second, no neuronavigation was available to determine the position of the TMS coil. However, we could demonstrate that rTMS clearly had an effect on working memory performance, which suggests that the TMS coil was correctly positioned according to the international 10–20 EEG system [30], [31]. On the other hand, the design of the study was such that working memory capacity was irrelevant for DG-P task performance; moreover, no significant correlation between working memory performance and economic decision-making was found. Arguably, however, we cannot rule out the possibility that for some reason the rTMS effect was stronger on the right, since digit span was reduced, relative to sham stimulation, only after rTMS to the right DLPFC, but not the left. Third, the DG-P as designed for this study does not allow to strictly distinguishing between a desire to punish unfairness and concerns for implementing equity. Along the same line, in light of the relatively low monetary gain for participants, it could be that the study design facilitated a tendency to induce fairness and to reduce selfish behavior. Future studies may consider more effective stimulation parameters to replicate the findings and to disentangle costly punishment from equity concerns more clearly.

In summary, the present study assigns a differential role to the DLPFC in economic decision-making that depends on individual personality traits and interpersonal attitudes. Whether or not people act in altruistic ways, it seems, is not necessarily a matter of empathy.

## Materials and Methods

### Ethics statement

The study was approved by the Ethics Committee of the Medical Faculty of the Ruhr-University Bochum. All participants gave written informed consent. The investigation was conducted in full accordance with the principles expressed in the Declaration of Helsinki.

### Participants

Twenty healthy subjects (13 women, seven men, *M*_age_ = 25.55 years, *SD*_age_ = 4.22 years) participated in this study. All subjects had normal or corrected-to-normal vision. They were recruited by advertisement from the Ruhr-University Bochum. For safety purposes all subjects had to complete a TMS-screening questionnaire [32] before participating in this study.

### Tasks

#### Dictator Game with Punishment Option

In the DG-P, subjects were told that they observed a scene between a proposer and a responder sharing 10 MU, and that they were in the position of a third-party punisher. Participants were instructed that such scenarios have been applied “online” in similar studies, but that the games were played offline. A structured debriefing after each session revealed that subjects had little difficulties in putting themselves into the shoes of a third-party and in appreciating that the scenarios depicted interpersonal behaviors that were very similar to “real-life” situations.

There were 11 trials per split-condition with shares of 5∶5, 7∶3, 8∶2, and 9∶1, respectively (thus 44 trials altogether). Participants were endowed with 10 MU per trial to invest in punishing unfair proposals. There was a ratio of 1∶2 concerning the punishment investment and the actual penalty. That is, for every 0.5 MU invested in punishment, the proposer's pay-off was reduced by 1 MU, and the recipient's pay-off was increased by the same amount. In other words, part of the game was the option not only to punish unfair proposers, but also to induce equity between the two players.

In each trial, participants first viewed pictures of two individuals who were going to share the money according to the offer of the proposer. The photographs were mainly selected from the Karolinska Directed Emotional Faces database (KDEF) [33], which may be used for non-commercial research purposes (http://www.emotionlab.se/resources/kdef). Since it is well known that physical attractiveness can influence decision making in economic games [34], [35], the stimulus material was rated for attractiveness on a seven-point Likert-type scale by 10 women and 10 men prior to the TMS experiment. None of these subjects participated in the TMS study. Only pictures of people with average attractiveness ratings (mean between 2.5 and 5.5) were used as stimuli. The gender ratio of the allocators and the recipients in the DG-P was counterbalanced. Next, participants saw the actual offer and were asked if they wanted to change the distribution between the players at their own expense. Thus, participants had the option to choose not to punish at all, or to invest as many MUs as they wanted to spend. Participants received 20 Euros for participation and received up to another 10 Euros depending on their behavior in the DG-P. That is, for each money unit invested (in Euros), participants received 10 percent in real money. For example, if a participant invested a total of 100 Euros in punishment over the three sessions (sham, right, and left rTMS), he or she actually received 10 Euros cash. Table 2 summarizes the total investment spent by each subject is shown for each stimulation condition.

**Table 2:** Total investment (standard deviation) in punishment for all unfair offers and empathy scores.

Subject	Total investment in punishment			Empathy (SPQ)
	Sham	rTMS to the right DLPFC	rTMS to the left DLPFC	
**1**	55.44 (8.35)	66.00 (9.46)	64.02 (8.75)	99.00
**2**	16.50 (5.50)	15.84 (5.51)	17.49 (4.98)	104.00
**3**	8.58 (0.78)	7.59 (0.50)	18.48 (5.96)	91.00
**4**	33.00 (5.50)	33.99 (5.51)	33.00 (5.50)	78.00
**5**	70.62 (8.84)	69.96 (8.11)	62.04 (6.59)	110.00
**6**	40.59 (5.64)	89.43 (9.74)	49.50 (5.50)	54.00
**7**	17.16 (5.51)	16.5 (5.50)	23.10 (4.65)	82.00
**8**	22.44 (2.78)	25.08 (4.35)	22.11 (4.55)	98.00
**9**	60.06 (8.35)	60.39 (8.40)	60.39 (8.40)	93.00
**10**	49.50 (5.50)	47.85 (4.75)	49.50 (5.50)	96.00
**11**	38.61 (8.40)	36.63 (7.33)	37.62 (8.35)	85.00
**12**	24.42 (4.06)	32.01 (5.01)	43.89 (5.72)	94.00
**13**	8.58 (4.89)	9.57 (5.06)	3.63 (2.03)	95.00
**14**	45.54 (12.08)	55.11 (12.76)	43.89 (10.92)	92.00
**15**	27.06 (7.22)	28.05 (8.10)	16.50 (5.50)	101.00
**16**	39.60 (11.05)	50.49 (7.98)	55.11 (8.40)	91.00
**17**	49.5 (5.50)	49.5 (5.50)	49.50 (5.50)	94.00
**18**	69.63 (8.78)	89.10 (9.55)	79.53 (12.51)	102.00
**19**	29.04 (4.03)	27.39 (3.70)	27.39 (2.26)	82.00
**20**	49.50 (5.50)	49.50 (5.50)	49.50 (5.50)	89.00

*Note*. The standard deviation represents the deviation across the three types of unfair offers.

**Figure 4:**
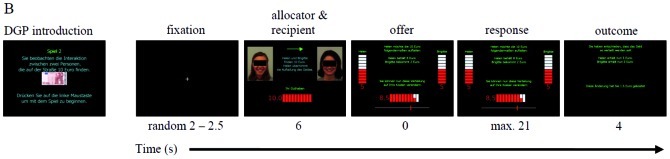
Introductory screen and stimuli in a single trial for the Dictator Game with punishment option. For publication purposes, the images were anonymized by superimposing black bars over the eye region. For further details, see the explanations in the text.

To minimize a possible overload on working memory due to mathematical operations, we visualized the outcome of punishment by depicting the participant's investment, the reduction of the proposer's money, and the recipient's gain simultaneously on slide bars. Participants were asked to confirm his or her final decision by pushing a mouse-button. Finally, participants were informed about the outcome of their decisions (Figure 4). Trials were separated by a fixation cross and presented randomly. Stimuli were delivered using Presentation® (Neurobehavioral Systems, Albany, CA). Across the three TMS conditions the DG-P took on average 11.22 min (*SD* = 0.91 min).

#### Digit Span

Since the exact duration of low-frequency TMS aftereffects is unknown, a working memory task was included in the experiment, based on previous findings showing that low-frequency rTMS over the right DLPFC disrupts digit span performance [30]. In other words, the task was included, in the absence of neuronavigation, to ensure that rTMS exerted a reliable effect on DLPFC function. Digit Span (DS) backward is considered to require an additional manipulation of items, whereas DS forward reflects short-term phonological storage and subvocal rehearsal. Therefore, both DS tasks were used to detect any effect. Starting with two, up to nine digits were read to the subjects and they had to repeat them immediately. There were two trials for each number of digits and subjects could advance to the next level only if at least one trial of the previous round was correct. Each correct trial counted as one point. Tthe maximum score for DS forward and backward was 16 points. DS was examined before each TMS session and immediately after completion of the DG-P.

### Empathy

The Saarbrücken Personality Questionnaire (SPQ) [36] is a slightly modified German version of the Interpersonal Reactivity Index [37]. The SPQ measures empathy on four dimensions, each comprising seven items to be rated on a five-point Likert scale, ranging from “strongly disagree” to “strongly agree”. The four dimensions are called “Perspective Taking” (spontaneous attempts to adopt the perspectives of other people), “Fantasy” (the tendency to identify with characters in fictional situations like movies), “Empathic Concern” (respondent's feeling of warmth, compassing, and concern for others), and “Personal Distress” (discomfort that results from observing another's negative experience). The psychometric properties of the SPQ have been repetitively examined. It has proven to be a reliable and valid instrument to examine empathy. The sum score reflects one's capacity to empathize with others (α = 0.85, maximum score 140, mean = 91.5, SD = 11.84).

### Repetitive Transcranial Magnetic Stimulation

A Medtronic MagPro R30 stimulator with MagOption (Medtronic Danmark A/S, Copenhagen, Denmark) and a Figure-8 coil (Model MCF-B65) with each wing measuring 8.5 cm were used to apply low-frequency (1 Hz) rTMS three times to 20 subjects. One pulse (280 ms) entails a maximum change of 32 kT/s of the electromagnetic field near the coil surface. Each subject received stimulation over the right DLPFC, the left DLPFC, and sham stimulation. The coil was fixed on a rack and placed tangential on the electrodes of the EEG cap. It was kept perpendicular to the underlying gyrus. Because neuronavigated positioning of the stimulus coil was not available, the electroencephalogram 10–10 coordination system was used to position the coil. In accordance with previous studies [14], [30] the right DLPFC was targeted by stimulating the cortex surface underneath the F4 EEG electrode and the left DLPFC underneath the F3 electrode. For sham stimulation the coil was tilted by 180° and held between Pz and Oz. Thus, subjects heard the same sound of clicking as during the real stimulation, however, there was no scalp sensation. To explain this perceptual difference between conditions, subjects were told that the stimulation of superficial nerves and muscles is the stronger the nearer the stimulation site is to the face, and that the scalp region under Pz is free of muscles. After the completion of all three sessions, participants were informed about the true reasons for the perceptual differences. A debriefing indicated that the subjects did not attribute the perceptual differences to a “fake” stimulation during the experiment.

Subjects received a 20-minute train (1200 biphasic pulses; intertrain interval of 1 s) with a stimulation intensity of 100% of the individual resting motor threshold (MT). During the 20-minute TMS subjects watched a neutral documentary movie. MT was defined as the lowest stimulation intensity that induced visible finger movements in at least three out of six trials when TMS was applied to the left motor cortex. The mean MT for the subjects was 54.55% (*SD* = 5.28%) of maximal stimulator output. All parameters were in accordance with the newest safety and ethical guidelines of TMS [38]. All subjects felt mild muscle contractions as side effects, and a few subjects described the procedure as slightly uncomfortable. One participant had chin twitches during the stimulation and two subjects reported mild headache after the experiment, one after stimulation of the right DLPFC and the other after sham stimulation.

### Procedure

The order of TMS conditions (inhibition of the right DLPFC, inhibition of the left DLPFC, and sham) was randomized across the three sessions. The mean duration between the first and the second session and between the second and the third one was 9.85 days (*SD* = 5.25) and 8.25 days (*SD* = 2.81), respectively. At the beginning of the first session subjects read a description of the experimental procedure. The functioning as well as possible side effects of TMS were explained. Subjects were seated approximately 80 cm in front of a computer monitor in a slightly reclined chair with a headrest. Prior to rTMS, subjects filled in the SPQ and demographic questionnaires.

### Statistical analyses

Behavioral data were compared by repeated-measures ANOVA as well as using a moderator analysis according to Judd et al. [24]. Simple planned contrasts were applied, if hypotheses about an effect were stated before the experiment. Partial eta-squared was calculated as a measure of effect size. All significance tests were two-tailed and P-values were set at .05. The normality of data distribution within groups and sphericity of repeated measures were assessed with Kolmogorov-Smirnov and Mauchly's tests, respectively. All variables were derived from normally distributed populations (all *p*s>.05). Where sphericity was violated, the degrees of freedom were corrected with the Greenhouse-Geisser estimates of sphericity. The relationships between psychometric and behavioral measures were analyzed with Pearson's correlation coefficients.
